# Measles lessons in an anti-vaccination era: public health is a social duty, not a political option

**DOI:** 10.1186/s13052-017-0420-6

**Published:** 2017-11-15

**Authors:** L. Lancella, C. Di Camillo, A. C. Vittucci, E. Boccuzzi, E. Bozzola, A. Villani

**Affiliations:** 1 General Pediatric and Infectious Disease Unit, Bambino Gesù Children’s Hospital, IRCCS, Piazza Sant’Onofrio 4, 00165 Rome, Italy; 2 Pediatric Emergency Department, Bambino Gesù Children’s Hospital, IRCCS, Piazza Sant’Onofrio 4, 00165 Rome, Italy

**Keywords:** Measles, Vaccination, Public health

## Abstract

**Background:**

Measles virus, member of the genus Morbillivirus in the family Paramyxoviridae, is a highly contagious human pathogen. An effective live-attenuated vaccine is available and its use has the potential to eradicate the disease from the human population. Although the vaccine was introduced in national vaccination schedules, several measles outbreaks have occurred because of insufficient vaccination coverage. Since early January 2017, a new outbreak of measles in Italy has been observed.

**Methods:**

We analyzed all the patients admitted to the Emergency Department of Bambino Gesù Children Hospital of Rome from the 1st of January 2017 to the end of May 2017 and discharged with diagnosis of suspected or confirmed measles or admitted to the Pediatric and Infectious Disease Unit. For each confirmed case, demographic data, vaccination history, exposure to source case, clinical presentation, date of onset of symptoms, hospitalization, laboratory test results, complications and therapy were collected.

**Results:**

From the 1st of January 2017 to the 31st of May 2017, we enrolled 139 patients who were conducted to the Emergency Department of Bambino Gesù Children’s Hospital because of measles: 33 patients were discharged with the diagnosis of suspected measles by clinical manifestations; 33 discharged with the diagnosis of confirmed measles by laboratory tests and 73 were admitted to the Pediatric and Infectious Disease Unit. Seven patients, who were exposed to mothers with measles, were admitted to receive treatment with Measles Immune Globulin intravenously. Among the 66 patients admitted to the hospital with measles, 31 cases (47%) occurred in unvaccinated individuals who were age-eligible for measles vaccination; 29 (44%) were infants too young to be vaccinated; only five patients (8%) received one dose of measles-containing vaccine.

Out of the 66 patients, 35 (53%) developed complications. Acute respiratory failure was the most reported complications (20%). Death, due to multiorgan failure by measles, occurred in one 9-girl-year-age patient with genetic disorders who was unvaccinated.

**Conclusions:**

Measles still represents a serious public health problem worldwide. Vaccination against measles is safe, effective, and cost-effective.

High vaccination coverage (>95%) with two doses of measles vaccine is crucial to elimination. Health care professionals play an important role in vaccination uptake and prevention of measles spread during an outbreak.

## Manuscript

Measles still represents a serious public health problem worldwide. It is a highly contagious infectious disease with severe complications: pneumonia, laryngotracheobronchitis, otitis media, diarrhea, hepatitis, keratoconjunctivitis, encephalitis, seizures and subacute sclerosing panencephalitis [1].

It is still responsible for more than 100,000 deaths every year [2]. An effective live-attenuated vaccine is available and its use has the potential to eradicate the disease from the human population [3].

Although the vaccine was introduced in national vaccination schedules 20 years ago, several outbreaks have occurred because of insufficient vaccination coverage in the European Region [4].

Since early January 2017, a new outbreak of measles in Italy has been observed: 2851 new cases were reported, 73% with age > 15 years and 27% pediatric patients; among those, 168 patients were <1 years old. Two hundred and twenty-four cases were healthy professionals. Most of the cases (89%) were unvaccinated; 6% received just one dose. Complications were reported by 35% of patients [5].

In Italy, measles is a disease subject to mandatory notification, and according to the routine procedure, physicians must report suspected measles case to their Local Health Units within 48 h of diagnosis. At the time of the study, vaccination for measles-mumps-rubella in a two-dose at 13–15 months and at 5–6 years of age respectively, is strongly recommended but not mandatory.

From the 1st of January 2017 to the 31st of May 2017, 139 patients (75 males, median age 3.82 years, range 16 days-20 years) were conducted to the Emergency Department of Bambino Gesù Children’s Hospital because of measles: 33 patients (24%) were discharged with diagnosis of suspected measles by clinical manifestations; 33 (24%) discharged with diagnosis of confirmed measles by laboratory tests and 66 (48%) were admitted to the Pediatric and Infectious Disease Unit.

Furthmore seven patients, six newborns and one unvaccinated 6 year old girl with metabolic disease, who were exposed to their mothers with measles, were admitted to receive treatment with Measles Immune Globulin intravenously (Flebogamma DIF 50 mg/ml). None developed measles.

For each patient admitted to the hospital, demographic data, vaccination history, exposure to source case, clinical presentation, date of onset of symptoms, hospitalization, laboratory test results, complications and therapy were collected.

Vaccination status was categorized as fully vaccinated, partially vaccinated, not vaccinated, too young to be vaccinated or unknown.

A source case was defined as a measles- infected individual who transmitted the disease to another previously uninfected individual.

Among the 66 patients admitted to the hospital with measles, confirmed by laboratory tests, (33 males; median age 3.36 years), the average duration of stay in the hospital was 7.42 ± 4.42 days (expressed as median ± SD). A confirmed case was defined either as a laboratory confirmed case in which measles-specific IgM antibodies were present in serum or in which measles virus nucleic acid was detected in urine or blood samples by PCR.

Thirty one cases (47%) occurred in unvaccinated individuals who were age-eligible for measles vaccination; of those, four had a chronic disease (one with Langerhans cell histocytosis, two with genetic disorders and one with cystic fibrosis). Twenty-nine (44%) were infants too young to be vaccinated; only five patients (8%) received one dose of measles-containing vaccine. For one patient, vaccination status was unknown. None were vaccinated with two doses.

In total, 42% of case patients had contact with a source case: of those, 46% were mothers with recent measles infection, who did not received vaccination.

Out of the 66 patients, 35 (53%) developed complications. Acute respiratory failure was the most reported complication (13 patients, 20%): 5% cases required intensive care. Six patients (9%) developed pneumonia. Death, due to multiorgan failure by measles, confirmed on post-mortem examination, occurred in one 9 year old girl with genetic disorders (dup7q36) who was unvaccinated. This kind of genetic disorder does not represent a condition preventing vaccination.

Encephalitis was verified in two patients. After discharge, the patients entered follow-up control; until now, no neuropsychological sequelae has been detected.

Other complications included laryngotracheobronchitis, neutropenia, thrombocytopenia, otitis, diarrhea and facial nerve paralysis. The characteristics of study population are represented in Table 1.

**Table 1:** The characteristics of study population

Characteristics	Number of cases
Patients	66
Male gender	33
Median Age (years)	3.82
Age group (months)	
1–15	29
> 15	37
Contact with source case	
Yes	27
No	39
Mothers with measles	13
Vaccination status	
Ineligible (aged <15 months)	29
No	31
Yes (I dose)	5
Unknown	1
Length of hospital stay (days)	7.42
Complications	35
Acute respiratory insufficiency	13
Hypertransaminasemia	7
Pneumonia	6
Encephalitis	2
Death	1
Others	13
Therapy	
Antibiotics	33
Measles Immune Globulin intravenously	2
Antiviral	2

From the 1st of January 2017 to the 31st of May 2017, Italy experienced high measles burden with 2851 cases reported to the measles surveillance system.

The present report analyses measles hospitalization rates in the pediatric population in Bambino Gesù Children’s Hospital of Rome over a 5-month period.

In our study, 53% of population developed complications. Acute respiratory failure was reported to be the most common cause of morbidity among the measles cases. Intensive care was required by three patients.

Death is reported in one 9 year old girl with genetic disorder who did not receive vaccination. This death serves as reminder that this particular category of children, the immune-compromised, is at elevate risk of severe measles complications.

Therefore it is essential to have a high vaccination coverage among them and their contacts.

One of the priorities of the health system in Italy is to control the diseases that are preventable through vaccination. For reaching the objective of measles elimination, the vaccination coverage of >95% with two doses of measles vaccine, is required [6]. In Italy, in 2016, vaccination coverage for measles was 87.26% at 24 months of age and 82.24% at 5–6 years [7]. These values are lower than they have been for over a decade and Italy remains one of the 14 countries in the European Region with ongoing endemic transmission [8].

Vaccines are among the most effective prevention tools available to clinicians. However, the success of an immunization program depends on high rates of acceptance and coverage. We believe that recent parental concerns about vaccine safety issues, such as a supposed association between autism and vaccines, though not verified by scientific evidence, had led increasing numbers of parents to refuse or delay vaccination for their children [9]. Furthmore parents are concerned that a child’s immune system could be weakened by too many vaccines, and they believe that it is better to develop immunity from natural disease than from vaccination; moreover they suppose that the severity of the disease, such as measles, is low [9]. Other groups of parents may refuse vaccination due to religious reasons or preference for complementary or alternative medicine. Finally some parents, prefer to obtain information from the Internet than to clinicians [10]. Many of the vaccine concerns identified among parents can be addressed through discussions with healthcare professionals and public vaccine information campaigns.

Out of the infected people, complications have been detected in 35% of patients, causing hospitalization and death. The decrease in measles immunization represents a problem for the whole Italian community, in particular for infants too young to be vaccinated or affected by immunodeficiency.

In our study, a substantial number of cases (44%) were in children less than 15 months of age not eligible for vaccination. In addition 46% of patients had been infected by mothers who were unvaccinated. Moreover infants of mothers who are not immune by natural infection become vulnerable at very early age and they represent a category at risk of developing severe or fatal complications [2].

It is necessary to identify susceptible individuals and consider undertaking catch-up immunization or supplementary immunization activities to close immunity gaps. Additionally measles immunization saves high costs for the national healthcare system [6]. In fact, measles is associated with direct cost (i.e hospital cost for complicated cases) and indirect cost (i.e. missing work days of parents).

Thus, we conclude that it is important to improve the knowledge and the scientific culture of healthcare professionals and all population for reaching adequate vaccination coverage for preventable diseases.
